# Complete chloroplast genome sequences of three aroideae species (Araceae): lights into selective pressure, marker development and phylogenetic relationships

**DOI:** 10.1186/s12864-022-08400-3

**Published:** 2022-03-19

**Authors:** Bicong Li, Tao Liu, Asjad Ali, Yao Xiao, Nan Shan, Jingyu Sun, Yingjin Huang, Qinghong Zhou, Qianglong Zhu

**Affiliations:** 1 Jiangxi Province Key Laboratory of Root and Tuber Crops Biology, Jiangxi Agricultural University, NO.1101 Zhimin Street Qingshanhu District, 330045 Nanchang, China; 2 Queensland Department of Agriculture and Fisheries, PO Box 1054, Mareeba, QLD 4880 Australia

**Keywords:** Aroideae, Chloroplast genome, Structural comparison, Selective pressures, Phylogenetic relationships

## Abstract

**Background:**

*Colocasia gigantea*, *Caladium bicolor* and *Xanthosoma sagittifolium* are three worldwide famous ornamental and/or vegetable plants in the Araceae family, these species in the subfamily Aroideae are phylogenetically perplexing due to shared interspecific morphological traits and variation.

**Result:**

This study, for the first time ever, assembled and analyzed complete chloroplast genomes of *C. gigantea*, *C. bicolor* and *X. sagittifolium* with genome sizes of 165,906 bp, 153,149 bp and 165,169 bp in length, respectively. The genomes were composed of conserved quadripartite circular structures with a total of 131 annotated genes, including 8 rRNA, 37 tRNA and 86 protein-coding genes. A comparison within Aroideae showed seven protein-coding genes (*accD*, *ndhF*, *ndhK*, *rbcL*, *rpoC1*, *rpoC2* and *matK*) linked to environmental adaptation. Phylogenetic analysis confirmed a close relationship of *C. gigantea* with *C. esculenta* and *S. colocasiifolia*, and the *C. bicolor* with *X. sagittifolium.* Furthermore, three DNA barcodes (*atpH-atpI* + *psaC-ndhE*, *atpH-atpI* + *trnS-trnG*, *atpH-atpI* + *psaC-ndhE* + *trnS-trnG*) harbored highly variable regions to distinguish species in Aroideae subfamily.

**Conclusion:**

These results would be beneficial for species identification, phylogenetic relationship, genetic diversity, and potential of germplasm resources in Aroideae.

**Supplementary Information:**

The online version contains supplementary material available at 10.1186/s12864-022-08400-3.

## Background

The subfamily Aroideae that consists of approximately 75 genera and over 1573 species with a type of inflorescence with spathe and spadix [1–3], is the largest and most diverse group of the family Araceae, which comprises 125 genera and about 3750 species [4]. The subfamily Aroideae is found mostly in the tropics and widely distributed in temperate zones [5], such as south and central America, New Zealand, southern China, South-east Asia, and west African, where various members of Aroideae perhaps show their importance in horticultural industry.

*Colocasia gigantea*, commonly known as Giant Elephant Ears, is a 150–300 cm tall perennial herbal plant with frost-tender boasting huge and heart-shaped green leaves up to 120–180 cm long and 90–150 cm wide. *Colocasia gigantea* is an important horticultural plant in the humid tropics and subtropics and used as a vegetable in many parts of South East Asia [6]. High dietary fiber and low sugar contents in its petioles make it attractive for diabetes and hypertensive patients [7].

*Caladium bicolor* is also known as caladiums, elephant ears, or angel wings. The *C. bicolor* is native to the open forests of tropical south America and typically grown for the bold and colorful foliage, the plant has a great ornamental value due to its multicolor foliage and has been cultivated in pots for indoor as well as lawn decoration [8]. All parts of the plant cannot be edible because of containing a mass of calcium oxalate and other toxic substances [9], but the leaf extracts possess antidiarrheal, anticonvulsant, anxiolytic and antidepressant properties [10].

*Xanthosoma sagittifolium* is known by various names such as malanga, cocoyam, tannia, arrowleaf elephant ears, and American taro [11]. The *X. sagittifolium* is native to tropical America but widely cultivated and naturalized in other tropical regions. The *X. sagittifolium* have sagittate leaves and commonly used as ornamental plants. It is also grown for the starchy corms and cooked as a popular regional dish (such as *fufu*) in west African tropical regions [12, 13].

Most of these herbaceous species in the Aroideae family (Araceae) that are used as foods and/or ornamentals belong to the genera *Colocasia*, *Caladium*, *Xanthosoma*, and *Alocasia*. However, similar phenotypic appearance and growth habits impede the identification, phylogenetic relationship, genetic diversity, and utilization of germplasm resources in Aroideae [1, 5, 14].

The chloroplasts play an important role in plant growth and development by conducting photosynthesis. The chloroplasts possess their own genetic material, a circular double-stranded DNA molecule, comprising of 110–130 genes (encoding ribosomal RNA, transfer RNA and proteins) ranging 107–218 kb in size [15]. Complete chloroplast genome (CPG) usually presents a highly conserved quadripartite structure consisting of a large (LSC) and a small single (SSC) copy regions separated by two inverted repeats (IRa and IRb). Comparing to nuclear genomes, chloroplast genome has a unique inherited model, a dense gene content and a slower mutation rate in evolution [16]. The CPGs are present in cells with higher number of copies that makes it favorable to use in terms of DNA extraction even from a small amount of sample including degraded ones [17]. Therefore, the CPGs have been recommended by the Barcode of Life Consortia as a molecular resource for developing molecular markers to genetically differentiate plant species [18]. Chloroplast-derived molecular markers have been widely used in taxonomic and phylogenetic researches, and provide many valuable information to resolve complex evolutionary relationships at multiple taxonomic levels [17, 19, 20]. With the development and application of high-throughput sequencing technologies in genome sequence, the high abundance of chloroplast DNA compared to nuclear DNA have made it relatively easy to obtain complete chloroplast genome sequence without prior purification of chloroplasts or its DNA [21]. Over 5000 complete chloroplast genomes sequences have been published from crop and other land plant genomes that lead to the development of comprehensive and accurate molecular markers for taxonomic, phylogenetic purposes and conservation of many valuable traits [22–24].

Comparing to the complex and huge nuclear genomes of Aroideae species [25], CPGs are smaller and easy to obtain, however, very limited genomic resources are available for subfamily Aroideae. Although the CPGs of several genera have been published [1, 26, 27], the phylogenetic relationship of Aroideae subfamily still needs attention specifically in areas of marker development and protein-coding gene selection. Therefore, further comprehensive studies on chloroplast genome resources with comparative analysis are necessary to solve these problems.

In the present study, we sequenced, de novo assembled and annotated the complete chloroplast genomes of *C. gigantea*, *C. bicolor* and *X. sagittifolium*. Furthermore, we compared the new chloroplast genome sequences of these three species with the published complete chloroplast genome sequences of 14 other Aroideae species. Our objectives were to: (1) to uncover Aroideae chloroplast genome and highly variable regions (hotspots) for developing molecular markers with high credibility; (2) to identify the protein-coding genes under selection that would play an important role in the adaptive evolution for Aroideae plants in ecosystems; (3) to construct a phylogenetic tree for locating the phylogenetic position of *C. gigantea*, *C. bicolor* and *X. sagittifolium*.

## Results

### General characteristics of three chloroplast genomes

**Fig. 1:**
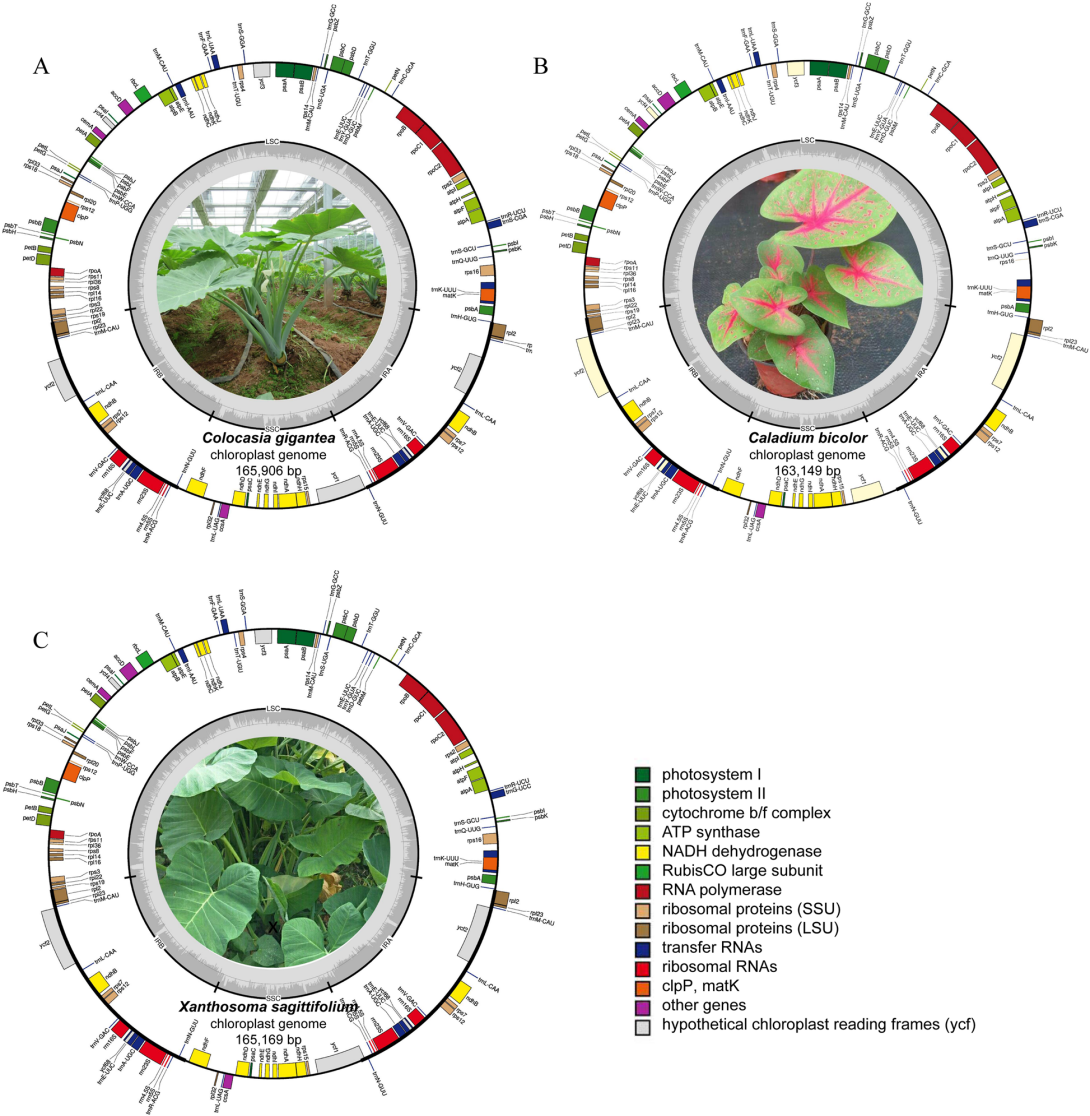
Chloroplast genome maps of *C. gigantea* (**A**), *C. bicolor* (**B**) and *X. sagittifolium* (**C**) with annotated genes. Genes inside the circle are transcribed clockwise, while those outsides are counterclockwise. Genes are color coded according to functional groups. Boundaries of the small single copy (SSC) and large single copy (LSC) regions and inverted repeat (IRa and IRb) regions are denoted in the inner circle for each species

The de novo assembly for the complete chloroplast genomes of *C. gigantea*, *C. bicolor* and *X. sagittifolium* produced 1,227,229, 792,949, and 1,109,410 clean reads with an average length of 150 bp with Illumina sequencing and filtering low-quality bases. The mean coverage of these reads on the chloroplast genomes of *C. gigantea* and *C. bicolor* was 1159 ×, 629 ×, and 927 ×, respectively, indicating the standard coverage of the reads enough to construct the complete chloroplast genome. The chloroplast genome lengths of *C. gigantea*, *C. bicolor* and *X. sagittifolium* were recorded as 165,906 bp, 163,149 bp, and 165,169 bp, respectively, followed by genome assembly and annotation steps (Fig. 1). All the CPGs displayed a typical quadripartite structure: one LSC region and one SSC region separated by two IR regions. The overall GC content percentages of *C. gigantea* (35.7%), *C. bicolor* (35.8%) and *X. sagittifolium* (35.7%) were similar among three genomes. The GC content of IR region (41.4–42.3%) of the four structural regions was significantly higher than that of the LSC (33.8–34.1%) region and SSC (28.6–29.4%) region for each CPG (Table 1). The three CPGs encoded an identical set of 131 functional genes including 86 protein-coding genes, 8 rRNA genes, 37 tRNA genes. Out of 131 genes, 17 were duplicated in the IR region, including 7 protein-coding genes, 6 tRNA genes, and 4 rRNA genes. Twenty-three genes had introns, including four genes (two rps12, one clpP and one ycf3) with two introns. The sequencing data of *C. gigantea*, *C. bicolor* and *X. sagittifolium* were deposited in GenBank under the accession numbers MN972442, MN972441, and MW628970, respectively.

**Table 1:** Whole genome sequence data and chloroplast genomes comparison of *C. gigantea*, *C. bicolor* and *X. sagittifolium*

species	*Colocasia gigantea*	*Caladium bicolor*	*Xanthosoma sagittifolium*
Locations	Jiangxi	Hainan	Guangxi
Whole genome reads	19,362,830	17,475,516	27,498,048
Chloroplast reads	1,227,229	792,949	1,109,410
Mean coverage	1159	629	972
Size (bp)	165,906	163,149	165,169
LSC (bp)	91,710	89,383	91,121
SSC (bp)	22,994	21,210	21,078
IR (bp)	25,601	26,278	26,485
Number of total genes	131	131	131
Number of CDS	86	86	86
Number of tRNAs	37	37	37
Number of rRNAs	8	8	8
IR duplication gene	17	17	17
Overall GC content (%)	35.7	35.8	35.7
GC content in LSC (%)	33.8	34.1	33.8
GC content in SSC (%)	28.6	29	29.4
GC content in IR (%)	42.3	41.5	41.4
GenBank number	MN972442	MN972441	MW628970

### Chloroplast genome size variation in Aroideae

**Fig. 2:**
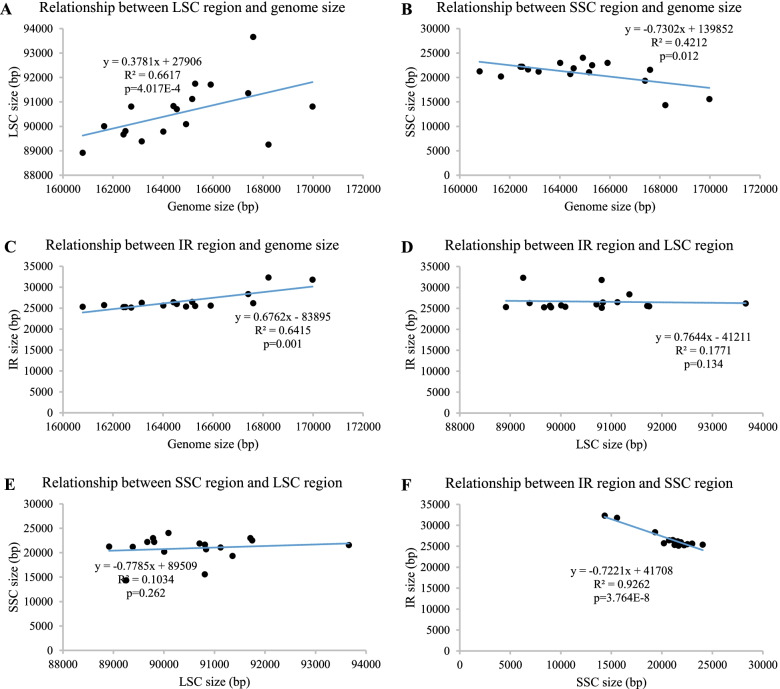
The correlational relationship among chloroplast genome size, LSC, SSC and IR regions (**A-F**)

Based on the complete chloroplast genome of *C. gigantea, C. bicolor* and *X. sagittifolium,* and 14 published CPGs, we conducted a comparative analysis on 17 CPGs in total. The CPG sizes in Aroideae ranged from 160,792 bp (*Arisaema ringens*) to 169,977 bp (*Typhonium blumei*), with an average CPG sequence length of 164,748 bp. All the CPGs displayed a typical quadripartite structure, the LSC length ranged from 88,915 bp (*A. ringens*) to 93,660 bp (*Arisaema erubescens*) with an average length of 90,568 bp, and SSC length ranged from 143,38 bp (*Carlephyton glaucophyllum*) to 24,044 bp (*Pinellia peltata*) with an average length of 20,925 bp. Two IR regions ranged from 25,131 bp (*Zomicarpella amazonica*) to 32,313 bp (*C. glaucophyllum*) with an average length of 26,627 bp (Table S1, Fig. S1). The overall chloroplast genome sizes showed a significant positive correlation with the LSC region (R^2^ = 0.662, *P* = 4.017E-4) and IR region (0.642, 0.001) (Fig. 2A, C), however, the SSC region was significantly negative in correlation with the overall genome sizes (0.421, 0.012) (Fig. 2B). It indicated the expansion of LSC and IR, and the contraction of SSC would promote the CPG size in Aroideae. In addition, the SSC was the only observed region with a significant negative correlation with IR region (0.9262, 3.764E-8) (Fig. 2F, D, E), suggesting a markable conflict between SSC and IR regions in Aroideae.

### IR/SC boundary and genome rearrangement

**Fig. 3:**
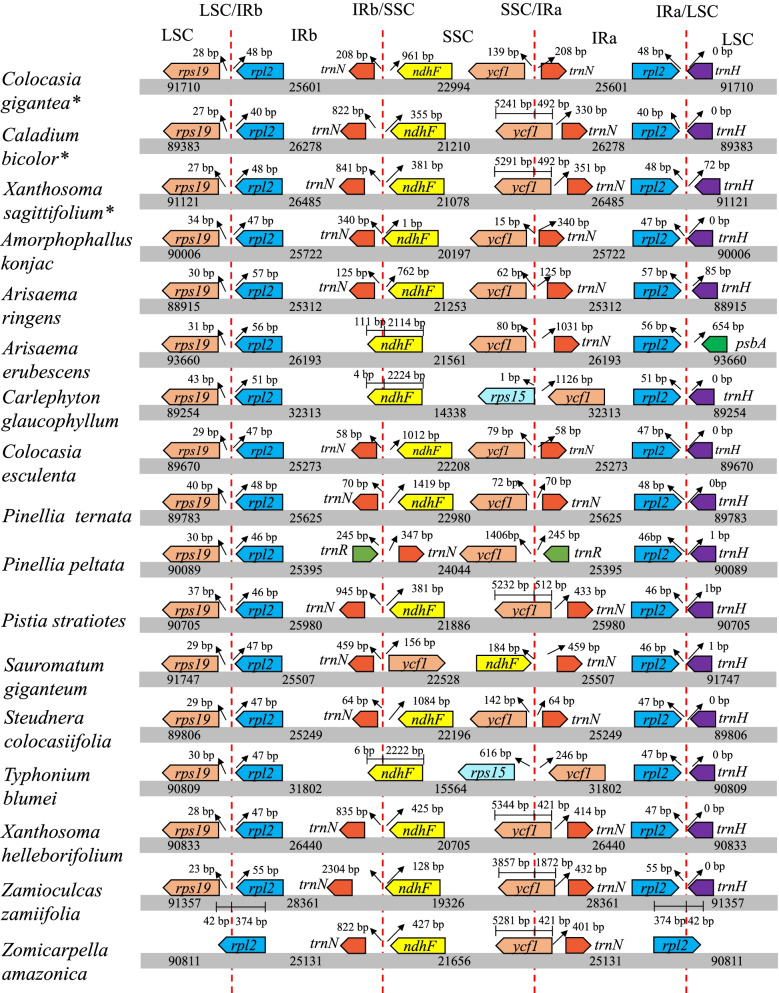
Comparison of border distances between adjacent genes and junction of the LSC, IR, and SSC regions among the 17 Aroideae chloroplast genomes. Number above the gene shows the distance between the ends of genes and the border sites. The figure is not to scale with respect to sequence length

The expansion and contraction of the IR and SC regions are the major causes of differentiation in chloroplast genome size and account for common evolutionary events in some families. To survey the variation of IR/SC boundary, a detailed comparison of the IR/SC boundary regions of *C. gigantea*, *C. bicolor* and *X. sagittifolium* with other 14 Aroideae species was conducted (Fig. 3). The LSC/IRb boundary was between or within rps19 and rpl2 with varying distances from the border in three types, while the IRa/LSC boundary was between rpl2 and trnH in all genera. However, based on the IRb/SSC and SSC/Ira differences, these chloroplast genomes could be divided into four types depending on the gene location in the IR/SC boundaries; type I contains the most species such as *C. gigantea*, *C. bicolor*, *X. sagittifolium*, *Amorphophallus konjac*, *A. ringens*, *A. erubescens*, *C. esculenta*, *P. ternate*, *Pistia stratiotes*, *Steudnera colocasiifolia*, *Xanthosoma helleborifolium*, *Zamioculcas zamiifolia* and *Z. amazonica*; type II occurred in *C. glaucophyllum*, *T. blumei*; type III and IV were present in only *Pinellia peltata* and *Sauromatum giganteum*, respectively. The IRb/SSC border in the type I was located within trnN and ndhF, the SSC/IRa in the type I were located within ycf1 and trnN, but the IRb/SSC border in the type II was located within ndhF, the SSC/IRa in type II was located between rps15 and ycf1. Our study showed the length of IRs in type I ranged from 25,131 bp to 28,361 bp, while the lengths of IRs in type II were 32,313 bp (*C. glaucophyllum*) and 31,802 bp (*T. blumei*), indicating the significant expansion of IR regions to merge more genes happened in type II and lead to duplication of ycf1. The IRb/SSC borders in type III were located between trnR and trnN, the SSC/IRa were located between ycf1 and trnN. Our results showed that the SSC length of *P. peltata* in type III was maximum in Aroideae, and the expansion of SSC regions included trnN, which lead to change the SC/IR boundary. However, the IRb/SSC borders in type IV were located between trnN and ycf1, the SSC/IRa in type III were located between ndhF and trnN. These results suggested that the SSC of *S. giganteum* chloroplast genome has been reverse complemented, which help in reverse the positions of genes at SSC region. All these events in the CPGs prove the expansion/contraction of two IR regions and the genome rearrangement.

### Sequence divergence analysis and nucleotide diversity

**Fig. 4:**
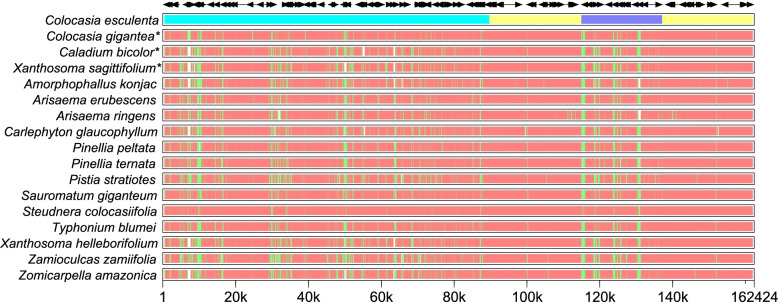
Structure comparison of seventeen chloroplast genomes using MultiPipMaker program. Black arrows and thick black lines above the alignment indicate genes with their orientation such as the cyan strip: LSC, yellow strip: IRs, blue strip: SSC, respectively. Pink strips represent different chloroplast genomes, green bars: mismatch and white bars: indel

**Fig. 5:**
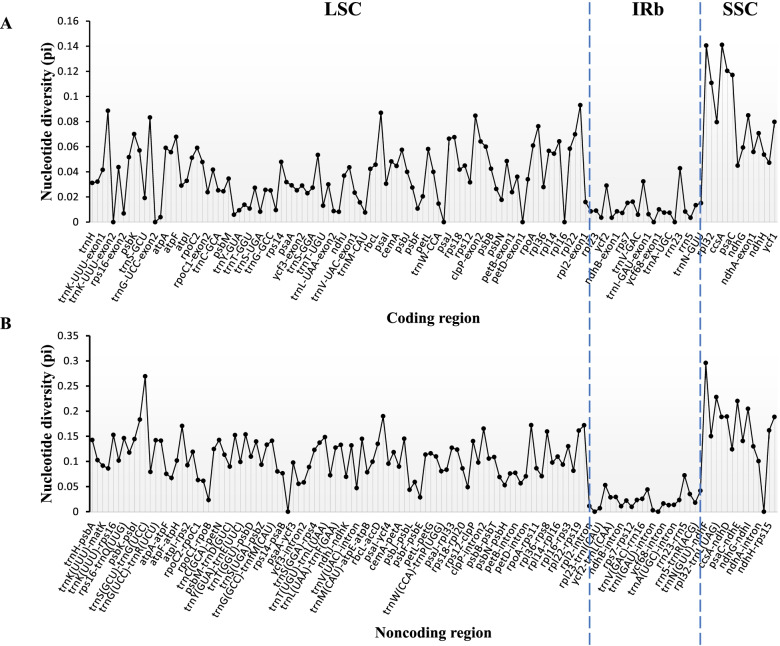
Comparison of nucleotide diversity (Pi) value for 130 coding regions and 131 noncoding regions among thirteen species in Aroideae

The CPGs of *C. gigantea*, *C. bicolor*, and *X. sagittifolium* were compared with other 14 species in Aroideae using MultiPipMaker software using *C. esculenta* as a reference. Two single-copy regions (LSC and SSC) were more divergent than two IR regions (Fig. 4), which might be the result of the four highly conserved rRNAs located in the IR region. Moreover, the data plot revealed that the noncoding region was more divergent than its coding counterparts (Fig. 5).

In order to confirm the sequence divergence and nucleotide diversity of different genome regions, the nucleotide diversity of 261 regions, including 130 protein-coding genes and 131 intergenic regions among the thirteen chloroplast genomes in Aroideae was analyzed using DnaSP software [28]. The results revealed that intergenic regions were more divergent than coding region (Fig. 5). The average nucleotide variability (Pi) in the noncoding regions was higher (0.099) compared to coding regions (0.038). The *trnN-ndhF* (0.295), *trnS-trnG* (0.269), and *rpl32-trnL* (0.228) intergenic regions were three top highest variables among the noncoding regions, while the genes *ccsA* (0.141), *ndhF* (0.140), and *ndhD* (0.121) were most variables among the coding regions. Several other highest-level divergences (Pi > 0.17) were found in the intergenic regions (*psaC-ndhE, ndhG-ndhI, accD-psaI, ccsA-ndhD, rps15-ycf1, trnL-ccsA, psbI-trnS, petD-rpoA, rps19-rpl2*, and *atpH-atpI*), and could be developed as specific molecular markers for species identification.

### Repeat analysis and simple sequence repeats (SSR) identification

**Fig. 6:**
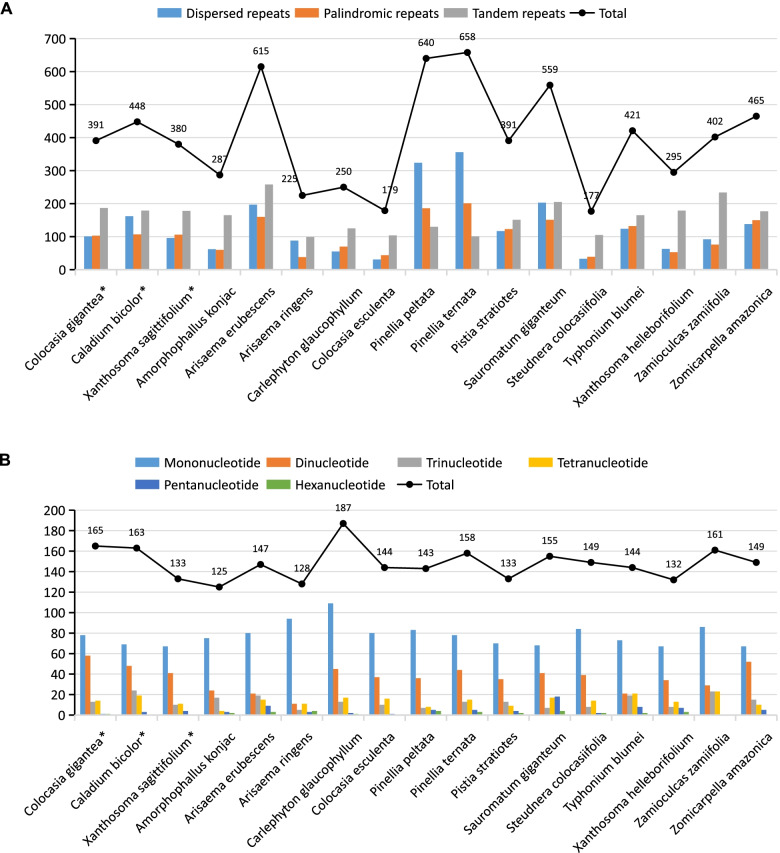
The type and presence of repeated units and SSRs in the chloroplast genomes of seventeen Aroideae species. **A** Number of three-types of repeats; **B** Number of SSRs and their types

Repeat units, distributed in the chloroplast genomes with high frequency, play an important role in genome evolution. The dispersed and palindromic repeat structures with length greater than 30 bp, and the tandem repeats greater than 7 bp in the seventeen species have been represented in the Fig. 6A. The repeats of the *C. bicolor* chloroplast genome consist of 448 total repeats, including 101 dispersed, 103 palindromic, and 187 tandems. However, *C, gigantea* and *X. sagittifolium* have smaller number of repeats, 391 and 380, respectively. Among the 17 Aroideae species, *C. esculenta* (179) had the lowest and *P. ternata* (658) had the highest number of repeats. Furthermore, we identified a total of 165, 163 and 133 SSRs by using MISA software within the chloroplast genomes of *C. gigantea*, *C. bicolor* and *X. sagittifolium*, respectively. The SSR number in the Aroideae species ranged from 125 (*Amorphophallus konjac*) to 187 (*C. glaucophyllum*) with an average number of 148 (Fig. 6B). The three focused species also had the SSRs near to the average value not the extreme value. The mononucleotide repeats in the chloroplast genomes of Aroideae species were most common (53%), followed by the dinucleotide repeats (25%), while the hexanucleotide repeats (1%) were the least. Most of the SSRs were located in the intergenic region of LSC, and the least amount of SSRs were in IR regions (Table S2). The 18 regions (*rps16-trnQ, trnS-trnG, atpH-atpI, rpoB-trnC, ycf3-trnS, trnT-trnL, trnF-ndhJ, rbcL-psaI, clpP-intron, rpl16-rps3, trnL-ndhB, trnN-ndhF, ndhF-rpl32, psaC-ndhE, ndhE-ndhG, ndhG-ndhI, rps15-ycf1,* and *ycf1*) contained more than three SSRs in at least one of the three species. Based on our results, there were only six regions (atpH-atpI, psaC-ndhE, trnN-trnF, trnS-trnG, ndhG-ndhI, rps15-ycf1) with high sequence divergence (Pi > 0.17) to be considered as the highly variable regions (HVR) for marker development and DNA barcode studies in Aroideae. Moreover, correlation analysis showed us that the dispersed, palindromic, tandem repeats, and SSRs have no contribution to the chloroplast genome size (Table S3).

### Selective pressure events

The ratio (ω) of 79 consensus protein-coding genes from 17 closely related species in Aroideae were calculated to estimate the selective pressure. Seven genes (*accD, matK, rbcL, rpoC1, rpoC2, ndhF, ndhK*) were found to experience positive selection by EasyCodeML software. The ω2 values (ω in M2a) ranged from 2.97 to 78.21, where rpoC1 with the highest ω2 value (78.21) in the M2a model. It suggested that *rpoC1* could be subjected to a significant positive selection. The consistent selective sites in these six genes were determined under naive empirical Bayes (NEB) and Bayes empirical Bayes (BEB) methods in M7 vs. M8 model. The results revealed that the gene *rpoC2* possesses 8 significantly positive selective sites, followed by *rbcL* (4) *rpoC1* (3), *matK* (2), *ndhK* (2) and *accD* (1), whereas no significantly positive selective site was observed in the *ndhF* (Table 2).

**Table 2:** The results of positive selective pressure analysis in M2a, M7 vs. M8 model

Gene name	Model	np	LnL	ω2(M2a)	LRTs(2ΔLnL)	LRT *p*-value	Positive sites
accD	M8	36	− 3560.21	4.58	7.44	2.42E-2	190 C*
	M7	34	− 3563.93				
matK	M8	36	− 3912.79	2.97	18.03	2.76E-4	314 Y *, 329 I*
	M7	34	− 3921.81				
rbcL	M8	36	− 3096.54	17.49	54.37	0	219 C**, 225 I*, 262 V**, 328 A**
	M7	34	− 3123.73				
rpoC1	M8	36	− 4177.33	78.21	96.64	0	91 Q**,150 C**, 436 K*
	M7	34	− 4225.65				
rpoC2	M8	36	− 9048.76	4.59	29.53	3.86E-7	80 L**, 533 K* 553 L*, 564 D*, 876 P*, 1025 S**, 1035 L*, 1356 L*
	M7	34	− 9063.53				
ndhK	M8	36	− 1464.89	5.06	15.38	4.57E-4	37 Q 0.963*,45 S 0.988*
	M7	34	− 1472.58				
ndhF	M8	36	− 5328.43	7.74	8.69	1.29E-2	
	M7	34	− 5332.78				

*: means *P* < 0.05, **: means *P* < 0.01

### Phylogenetic analysis

**Fig. 7:**
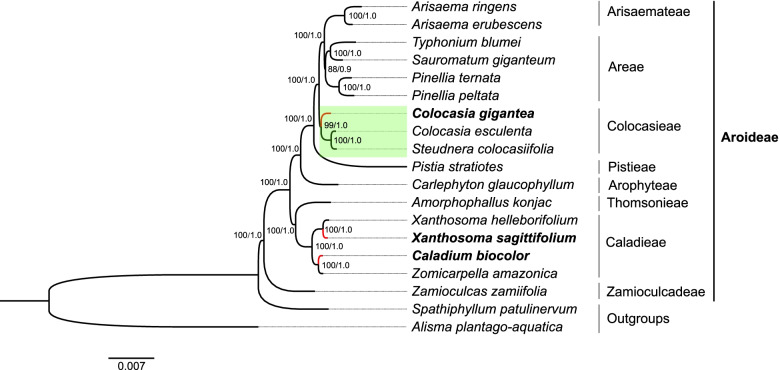
Phylogenetic relationship of the 19 species inferred from ML and BI analyses based on the 79-consensus protein-coding sequences. The bootstrap values of ML and Bayesian posterior probabilities of BI analyses are shown beside the node of clades. *S. patulinervum* and *A. plantago-aquatica* was used as the outgroups. *C. gigantea, C. bicolor* and *X. sagittifolium* were marked in bold characters and red branches

In order to identify the phylogenetic positions of the *C. gigantea*, *C. bicolor* and *X. sagittifolium* within the subfamily Aroideae, we utilized different regions, including the complete chloroplast genome, LSC, SSC, IR, and 79 consensus protein-coding sequences of 19 species to construct the phylogenetic tree using *Spathiphyllum patulinervum* and *Alisma plantago-aquatica* as the outgroups (Fig. 7, Fig. S2). We found that the phylogenetic tree based on the CDS have high bootstrap values (> 75) in maximum likelihood (ML) analysis and were strongly supported by greater than 0.9 posterior probabilities in Bayesian inference (BI) analysis, whereas the phylogenetic trees constructed from CPG, LSC and IR have different topology with relatively lower bootstrap values and posterior probabilities, suggesting that the phylogenetic tree constructed from CDS and SSC regions have higher credibility than the phylogenetic trees constructed from CPG and IR. Furthermore, the CDS phylogenetic tree confirmed that the three species belong to the subfamily Aroideae. The *C. gigantea* was closer to *C. esculenta* and *S. colocasiifolia.* Similarly, the species *X. sagittifolium* and *X. helleborifolium* clustered into a paraphyletic group with the *C. bicolor* and *Z. amazonica*, respectively. Furthermore, *S. colocasiifolia* was observed near genus *Colocasia* in our various phylogenetic trees.

### Molecular marker development

**Fig. 8:**
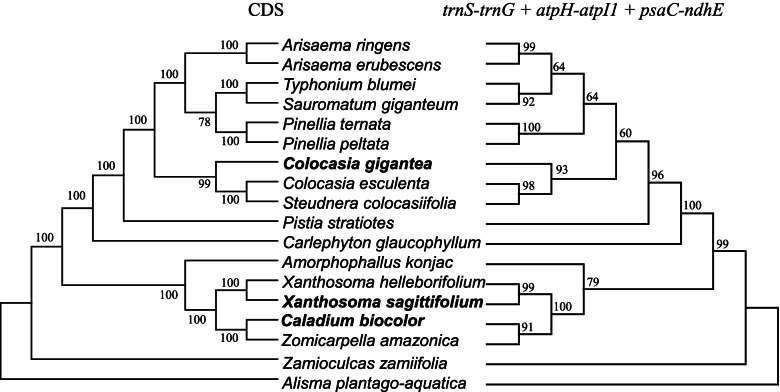
Phylogenetic tree for 17 Aroideae species using the CDS of 79 protein-coding genes and *atpH-atpI* + *psaC-ndhE* + *trnS-trnG* DNA barcode combinations

A comprehensively comparative analysis on nucleotide diversity and SSRs resulted in the six regions (*atpH-atpI*, *psaC-ndhE*, *trnN-trnF*, *trnS-trnG*, *ndhG-ndhI*, *rps15-ycf1*) with high nucleotide diversity and possibility of developing more than three SSRs as candidate DNA barcode for molecular markers. The comparative analysis of these new markers (Table 3) showed that the *atpH-atpI* have highest discrimination success (94%) within the six candidate DNA barcodes followed by *trnS-trnG* (83%) and *psaC-ndhE* (77%) compared to low discrimination success of *ndhG-ndhI*, *ps15-ycf1*, and *trnN-ndhF*. We didn’t observe any single candidate DNA barcode with 100% discrimination success. Three regions (*atpH-atpI*, *psaC-ndhE*, *trnS-trnG*) with discrimination success ratios > 75% were combined as new candidate DNA barcode. These three combined markers (*atpH-atpI* + *psaC-ndhE*, *atpH-atpI* + *trnS-trnG*, *atpH-atpI* + *psaC-ndhE* + *trnS-trnG*) showed 100% discrimination success, especially, the phylogenetic tree constructed from *atpH-atpI* + *psaC-ndhE* + *trnS-trnG* with high credibility (bootstrap value > 60), could be developed as an accurate molecular marker in Aroideae (Fig. 8).

**Table 3:** Comparative analysis of the new markers in Aroideae

Makers	Alignment length (bp)	Variable sites (%)	Information sites (%)	Number of bootstra*p* values > 75	Discrimination success ratio (%)
*atpH-atpI*	1330	398 (29.9)	102 (7.7)	11	94
*trnS-trnG*	3475	1563 (45.0)	734 (21.1)	6	83
*psaC-ndhE*	1773	844 (47.6)	343 (19.3)	6	77
*ndhG-ndhI*	1350	560(41.5)	220(16.3)	0	66
*rps15-ycf1*	2160	944(43.7)	439(20.3)	0	55
*trnN-ndhF*	9621	2955(30.7)	1015(10.5)	3	38
*atpH-atpI* + *psaC-ndhE*	3103	1242 (40.0)	445 (14.3)	9	100
*atpH-atpI* + *trnS-trnG*	4805	1961 (40.8)	836 (17.4)	11	100
*psaC-ndhE* + *trnS-trnG*	5248	2407 (45.9)	1077 (20.5)	9	94
*atpH-atpI* + *psaC-ndhE* + *trnS-trnG*	6578	2805 (42.6)	1179 (17.9)	12	100

## Discussion

In this study, the complete chloroplast genomes of three species of subfamily Aroideae were assembled using Illumina sequencing technology followed by a comparative analysis, all methods were carried out in accordance with relevant guidelines and regulations. A good level of similarity was observed among three genomes in terms of genome structure, gene content and gene arrangements, however the chloroplast genome of *C. gigantea* showed differences with *C. bicolor* and *X. sagittifolium* in SSC/IR boundary, and *C. bicolor* and *X. sagittifolium* in terms of the expansion of IRs to merge part of *ycf1* (Fig. 3). Similar structural variation was found in 14 species of Aroideae, including *P. stratiotes*, *X. helleborifolium*, *Z. zamiifolia* and *Z. amazonica*. Notably, the complete *ycf1* region was included in the IR of *C. glaucophyllum* and *T. blumei*, and a significant correlation between CPG size and IR size was observed. These results indicated that most of the variations in chloroplast genome structure occur due to the contraction and expansion of IR region [19].

The comparison of the chloroplast genome sequences obtained from sequence divergence analysis in this study showed us clear differences between these species at the molecular level of CPG. The intron region showed the highest variable rate, followed by the SSC, LSC, protein-coding regions, and IR region with the having the smallest rate. Our results are consistent with the previous studies on the chloroplast genomes of many land plants [15, 24, 29]. The nucleotide diversity of noncoding regions was higher than that in coding regions, suggesting suitability of the noncoding regions in Aroideae for the molecular marker identification, this is consistent with previous research in angiosperm chloroplast genomes [29], Thirteen intergenic regions (specifically trnS-trnG) with highest-level of divergences (Pi > 0.17) could be developed as specific molecular markers for species identification [30]. Similarly, *psaC-ndhE*, *trnN-ndhF*, *ccsA-ndhD*, *rps15-ycf1*, *petD-rpoA*, *atpH-atpI*, *rpl32-trnL*, *rps19-rpl2*, *trnL-ccsA* have been reported for the discrimination of potential molecular markers and DNA barcodes [15, 29, 31]. The six highly variable regions (*atpH-atpI*, *psaC-ndhE*, *trnN-trnF*, *trnS-trnG*, *ndhG-ndhI*, *rps15-ycf1*) contained at least three SSRs in *C. gigantea*, *C. bicolor* or *X. sagittifolium* (Table S2). Previously, highly variable regions have been compared for whole-genome sequences in Rosaceae and indicated as hotspots in positive correlation with the distribution of SSRs [15]. These results would improve our understanding of chloroplast genome of Aroideae by the repeats identification and nucleotide diversity analysis.

Analysis of the adaptive evolution of genes has an important reference value in examining the change of gene structure and functional mutations. The KA/KS ratio may reveal the constraints of natural selection on organisms, and the estimation of these mutations contribute greatly in understanding the dynamics of molecular evolution [19, 29, 32]. In the present study, there were seven genes (*accD, ndhF, ndhK, rbcL, rpoC1, rpoC2, matK*) under positive selection with significant selective sites. Among these, the *accD* gene encodes the β-carboxyl transferase subunit of acetyl-CoA carboxylase [33], which is an important regulatory enzyme for fatty acid synthesis. The *accD* has been reported as an essential gene required for leaf development [34], and as a contributor in leaf longevity [35]. Considering the fact that Aroideae species commonly have large leaf area, the finding of the *accD* under positive selection might indicate that it is an essential factor for leaf development. Similarly, *rpoC1* and *rpoC2* encodes the RNA polymerase β, which might play an important role in the regulation of pollination and sex differentiation [29]. The *matK* encodes an intron maturase (maturase K) which is involved in the cutting/splicing of Group II RNA transcriptional introns [36]. Furthermore, three other genes (*ndhF, ndhK*, and *rbcL*) under positive selection showed photosynthesis linked roles, indicating their role in photosynthesis and carbon fixation in Aroideae. These genes (*accD*, *rbcL*, *ndhK*) to have been reported to undergo positive selection in the Monsteroideae (Araceae) [32]. The Aroideae species have diversity of the ecological niches, and most of the species in the Aroideae are distributed in tropical humid forest, such as swamps, river margins and damp sites [37]. Therefore, chloroplast functional genes, involved in energy metabolism and plant development, might play key roles during the adaptation and development of the Aroideae species to their respective ecological niches.

Based on similar morphological characteristics and the lack of nuclear genome information, defining the phylogenetic relationships in Aroideae is an important and difficult goal to reach [1]. Phylogenetic analysis using the chloroplast genome sequence has been applied to evaluate evolutionary relationships of species [17, 19, 20]. Complete chloroplast genome sequence would be a great molecular resource for exploring phylogenetic relationships compared to whole nuclear genome in Aroideae and its relative species [1, 32, 38–40]. Phylogenetic tree constructed in this study based on complete chloroplast genome, CDS, LSC, SSC, IR, and intergenic regions, showed results in consistence with the traditional classification system [3, 41–43], indicating the rational of the classification of Aroideae. The shape, size and color of leaf and petiole of *C. gigantea* are similar to *X. sagittifolium*, and *C. bicolor* have markable differences of leave size and color with *X. sagittifolium* (Fig. 1). However, it is not easy to reveal their phylogenetic relationships as their looks. *Colocasia*, *Caladium* and *Xanthosoma* were taxonomically assigned to Colocasieae / Colocasioideae based on available phenetic data in previous researches [2, 44], whereas follow-up studies showed that *Caladium* and *Xanthosoma* would be in Caladieae / *Amorphophallus* clade, and *Colocasia* would be in *Colocasia* / *Pistia* clade, speculated from phylogenetic analyses based on the data of organelle DNA sequences, restriction-sites, morpho-anatomy, and fossils [3, 41–43]. Our phylogenetic analysis based on complete chloroplast genome further support these traditional classification by assigning *Colocasia*, *Caladium* and *Xanthosoma* into different clades, *C. gigantea*, *C. esculenta* and *S. colocasiifolia* were group into a monophyletic group, the *S. colocasiifolia* is nested in the *Colocasia*’s clade, the resemble results have been in previous researches [45], the *C. bicolor* and *X. sagittifolium* compose a paraphyletic group with *Z. amazonica* and *X. helleborifolium* in Caladieae clade. Other 10 species in Aroideae have been classed into the corresponding phylogenetic position as previous researches (Fig. 7) [3, 41–43], indicates the complete chloroplast genetic information have fine reliability to better understand the phylogenetic relationships in Aroideae.

Accurate discrimination of germplasm is very important for its utility, breeding new cultivars and evolutionary relationships [46]. Discrimination based on only morphological traits in Aroideae would not provide the complete picture of the family unless combined with the DNA markers. Previously, researchers focused on mutational and evolutionary dynamics in chloroplast genome of Aroideae [1, 26, 27], however, development and application of DNA barcodes have been rarely reported. DNA barcodes are defined as the DNA sequences with a high mutation rate to identify a species within a family [47]. Plastid (chloroplast) genome have such hotspot regions to be used as DNA barcodes for identification purposes in closely related species [18, 47]. Here, three candidate DNA (highly variable regions) barcodes such as *atpH-atpI, psaC-ndhE, trnS-trnG* were detected (Fig. 8, Table 3), in order to validate the discrimination effect of these molecular markers, the combined DNA barcodes of *atpH-atpI* + *psaC-ndhE* + *trnS-trnG* were manually extracted from other 13 published chloroplast genomes of Aroideae spesies [1], the phylogenetic tree contained 30 Aroideae species and *Alisma plantago-aquatica* was analysed (Fig. S3), and the relationships among these species in the phylogenetic tree were almost consistent with the previous taxonomic structure [1]. As our results showed, most of the candidate DNA regions are in LSC region and these regions can discriminate Aroideae species successfully when used in combination forms. Similar results were reported for chloroplast genomes of Oryza [16], Cucurbitaceae [29] and Rosaceae [15]. Therefore, these variable regions could be employed as specific DNA barcodes for identification purposes and genetic diversity studies in subfamily Aroideae.

## Conclusion

Present study reported the complete chloroplast genomes of *Colocasia gigantea, Caladium bicolor* and *Xanthosoma sagittifolium,* which provided valuable resources to understand subfamily Aroideae. Seven protein-coding genes (*accD, ndhF, ndhK, rbcL, rpoC1, rpoC2, matK*) were found to undergo selection, which might be the result of adaptation to the environment. Phylogenetic relationship analysis revealed that the *C. gigantea* was sister to *C. esculenta* and *S. colocasiifolia*, the *C. bicolor* was closer to *X. sagittifolium* compared to *C. gigantea*, and *S. colocasiifolia* would be belonged to the genus *Colocasia*. Furthermore, several highly divergent noncoding regions were identified that would be beneficial for developing high-resolution molecular markers. And newly developed DNA barcodes presented a solid resource to distinguish the Aroideae species and study phylogenetic relationships.

## Methods

### Plant materials and DNA extraction

The fresh and healthy leaves of *C. gigantea*, *C. bicolor* and *X. sagittifolium* were collected from adult plants growing for 3 months in the Araceae resource nursery of Jiangxi Agricultural University (Jiangxi, China) and frozen at − 80 °C until further use. Three voucher specimens were collected from Jiangxi (*C. gigantea*, T2–31), Hainan (*C. bicolor*, T3–37) and Guangxi (*X. sagittifolium*, T5–34) province of China with permission and deposited in the Tuber Crop Genetic Research Laboratory of Jiangxi Agricultural University. Total genomic DNA was extracted from ~ 200 mg sample using modified CTAB protocol. DNA quality and integrity were assessed in a Nanodrop 2000 spectrophotometer and evaluated using a 0.8% (w/v) agarose gel. The other eleven published complete chloroplast genomes were retrieved from the National Center of Biotechnology Information (NCBI) for conducting the follow-up analyses.

### Illumina sequencing, assembly, and annotation

DNA sample of three species were used to build paired-end libraries with average insert size of 500 bp and sequenced using an Illumina Hiseq 2500 platform (BGI, Tianjing, China) followed by filtering of poor-quality raw reads using Trimmomatic software. It resulted in the form of 1–2 Gb of sequence data after base quality control, which was deposited in the China National GeneBank (CNGB) under project CNP0001850. The chloroplast genome of *C. gigantea* and *C. bicolor* was assembled by using SPAdes (v 3.12.0) [48], BlastN (v2.7.1), and Gapcloser (v1.12-r6). Firstly, these reads were assembled by using the Plasmidspades.py in SPAdes. Secondly, Contigs representing the chloroplast genome were retrieved, ordered, and incorporated into a single draft sequence by comparing with the chloroplast genome of *Colocasia esculenta* (NC_016753.1) using BlastN. Thirdly, the gaps in the chloroplast single draft sequence were removed by using GapCloser. Finally, the complete genome sequence was annotated by the combined results from CPGAVAS2 [49] and GeSeq [50] followed by manual corrections of the positions of the start and stop codons and the intron/exon boundaries by Blastp against the GenBank database. The circular chloroplast genome maps were drawn using the online program OGDRAW [51]. The three newly generated complete chloroplast genome sequences were validated and submitted to GenBank by using Sequin.

### Whole chloroplast genomes comparison

In order to better discover the intergeneric variation among the complete chloroplast genome sequences by genomes comparison in the subfamily Aroideae, 14 published complete chloroplast genomes were compared. The details of the species are provided in Table S1. We used MultiPipMaker program with default parameters to compare and visualize the alignments [52] by using reference CPG of *C. esculenta*. The IR region borders and gene rearrangements were surveyed by manual inspection to analyze the expansions, contractions, and variation in junction regions among 17 Aroideae species. The bivariate correlational relationship between the overall CPG sizes and each of the structural regions of CPGs (LSC, SSC and IR) were analyzed by SPSS v19.

### Repeated sequences identification

A sequence search for four types (dispersed, palindromic, tandem, and microsatellite repeats) of repeated sequences was conducted in all 17 species. An online program Vmatch was used to search out the size and location of dispersed and palindromic repeats with parameters of 30 bp minimal repeat size, the similarity percentage (at least 90%) of two repeat copies followed by manually filtering the redundant output of Vmatch by merging overlapping repeats into one repeat motif whenever possible. The tandem repeat sequences at least 7 bp in length was detected by the online program Tandem Repeats Finder with the alignment parameters for match, mismatch, and indels set at 2, 7, and 7, respectively. Microsatellites (SSRs) were searched by MISA with the parameters set as the thresholds of 10, 5, 4, 3, 3, and 3 for mono-, di-, tri-, tetra-, penta-, and hexa-nucleotide, respectively.

### Sequence divergence and selective pressure analysis

To analyse the sequence divergence of the chloroplast genomes in Aroideae family, the nucleotide variability (Pi) of the gene-coding regions and intergenic regions was analyzed using DnaSP (v 6.12.03) based on the method of Shi et al. (2019). Selective pressure was analyzed for consensus protein-coding genes among 17 genomes from Aroideae species. Easy-CodeML software with the site model with four comparison models (M0 vs. M3, M1a vs. M2a, M7 vs. M8 and M7a vs. M8a, LRT threshold *p* < 0.05) was used to calculate the nonsynonymous (Ka) and synonymous (Ks) substitution ratios and likelihood ratio tests (LRTs). The values of both Ka/Ks (ω) and the LRTs were coupled to evaluate the selection on amino acid sites [53].

### Phylogenetic relationships

To reconstruct the phylogenetic relationships and confirm the phylogenetic position of the *C. gigantea*, *C. bicolor* and *X. sagittifolium*, 17 CPGs including 14 published CPG sequences from Aroideae were aligned using the software MAFFT v7.017, *S. patulinervum* and *A. plantago-aquatica* were used as the outgroups. Because the different CPG regions have the differentiation of the molecular evolutionary rate, phylogenetic relationship analyses were performed using the following five datasets: (1) the overall CPG sequences; (2) LSC; (3) SSC; (4) one inverted repeats region; and (5) consensus protein coding genes (CDS). The best-fitting nucleotide substitution model for each dataset based on the Akaike information criterion (AIC) was determined by Modeltest 3.7 [54]. The phylogenetic trees were constructed using MEGA-X [55], and a bootstrap test was performed with 1000 repetitions to calculate the maximum likelihood (ML) bootstrap value with Tamura-Nei model. Bayesian inference (BI) analysis was conducted using MrBayes 3.2.3 with Markov Chains Monte Carlo (MCMC) to estimate posterior probability distributions [56], the GTR + I + G model was set in MrBayes, the simulations algorithm for 1000,000 generations with four incrementally heated chains, starting from random trees, and sampling trees every 1000 generations, the first 250 generations (25% of trees) were discarded as burn-in. The phylogenetic trees were visualized using Figtree (v1.4.3).

### Molecular marker development

The sequence regions on the CPG with high nucleotide diversity and over three SSRs were selected as the candidate DNA barcode. Each candidate DNA barcode was used to construct phylogenetic tree for validating its efficiency, the alignment length, variable sites, information sites and bootstrap values using MEGA software. The discrimination success resulted from the comparation with the phylogenetic tree construct from candidate DNA barcode and all protein-coding gene sequences with the most credibility in this study.

## Supplementary Information

**Additional file 1.**

**Additional file 2.**

## Data Availability

The genome raw reads have been deposited in the China National GeneBank (CNGB, https://db.cngb.org/) under a Project accession: CNP0001850. The three complete chloroplast genomes (MN972441, MN972442, MW628970) have been deposited and available in the National Center for Biotechnology Information (NCBI, https://www.ncbi.nlm.nih.gov/). The materials are available from the corresponding author on reasonable request after the publication of the work.
