# Supplementary material for: Complete chloroplast genome sequences of three aroideae species (Araceae): lights into selective pressure, marker development and phylogenetic relationships
Source: BMC Genomics. 2022 Mar 19;23:218. doi: 10.1186/s12864-022-08400-3 (PMC8933883; doi:10.1186/s12864-022-08400-3)
Supplement: Supplementary file 2 — Additional file 2. [file 12864_2022_8400_MOESM2_ESM.docx]

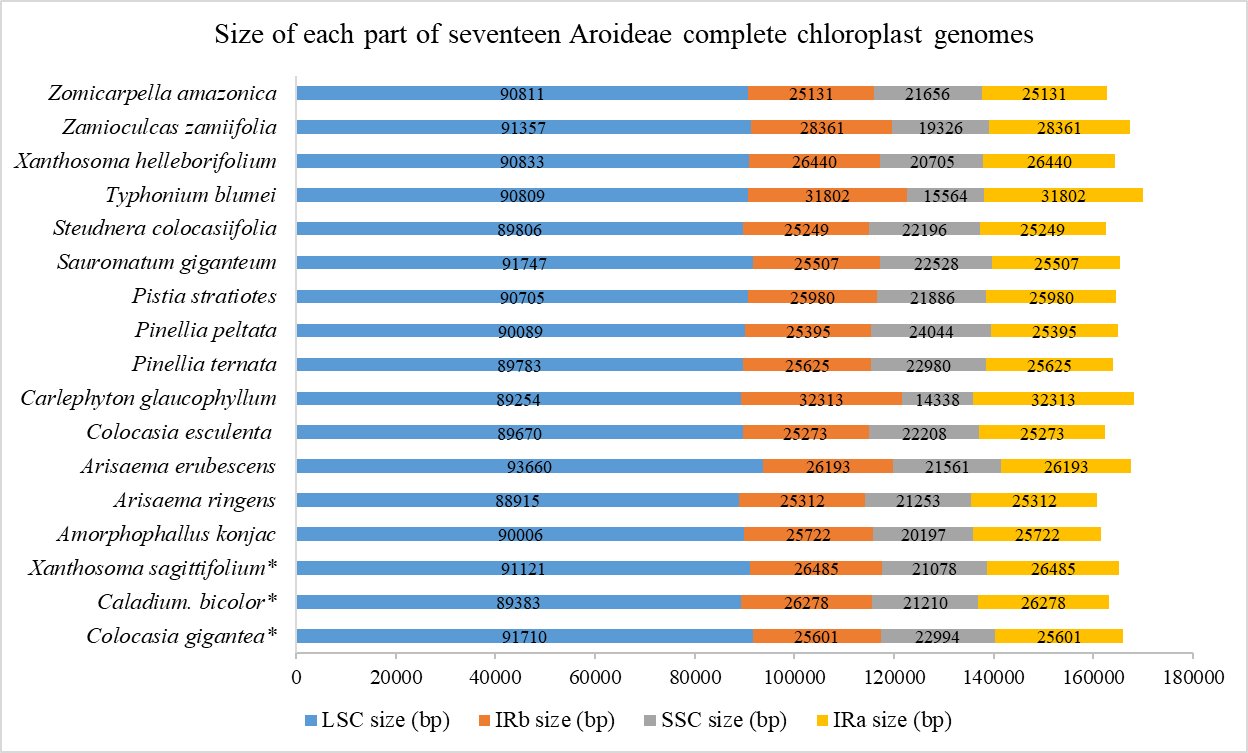


**Fig. S1**. The sizes of each part of complete chloroplast genomes of seventeen Aroideae species.


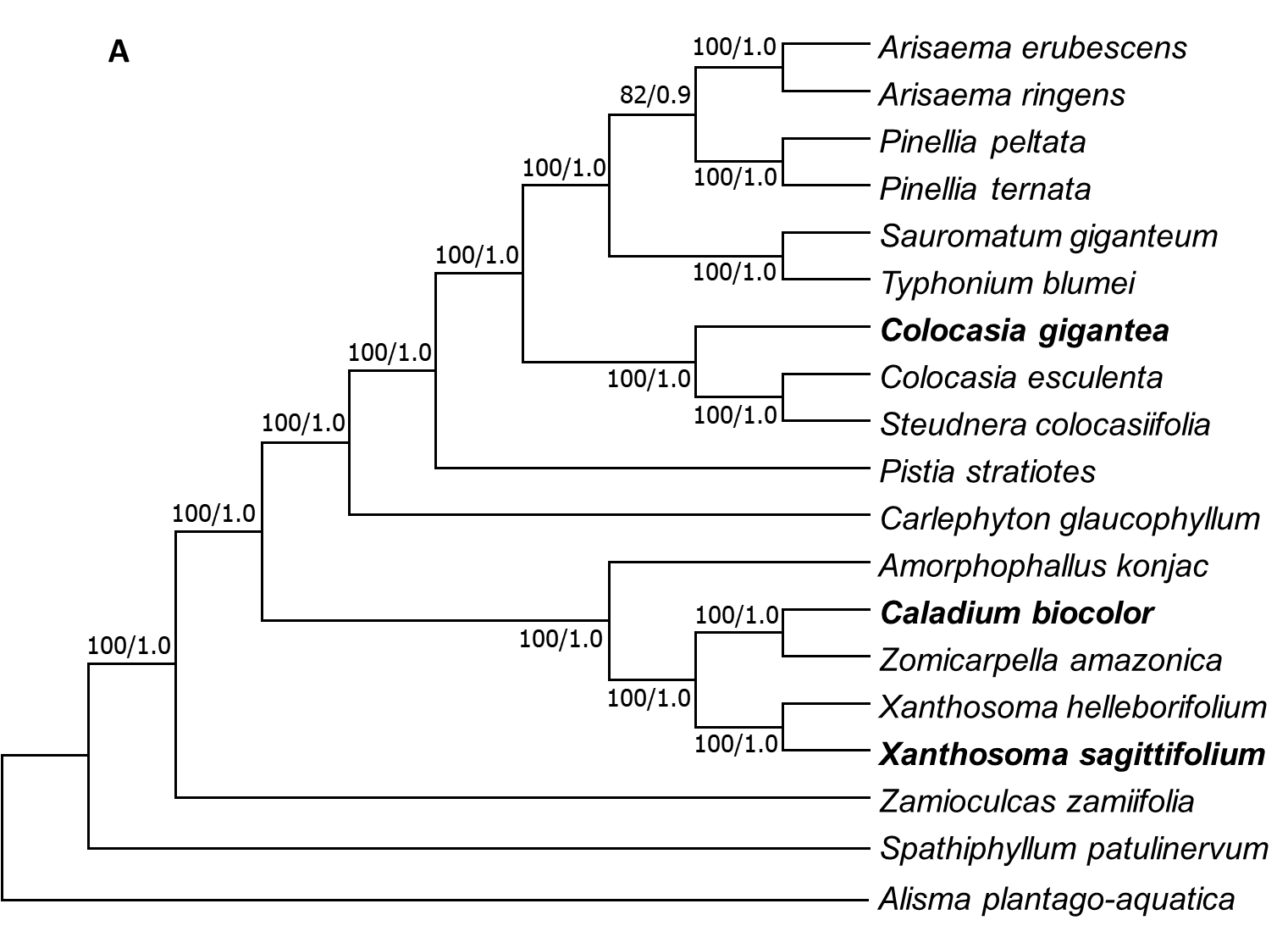

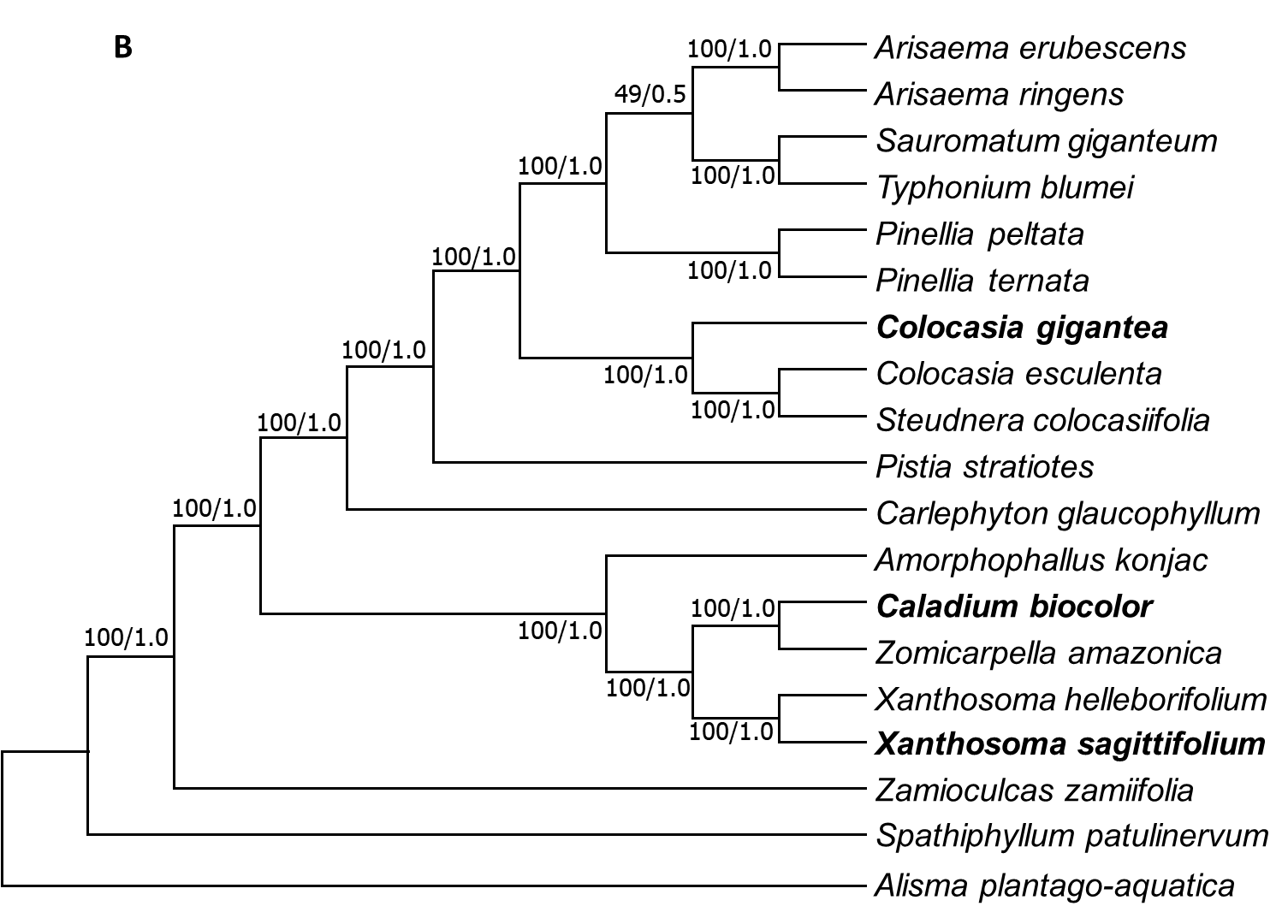


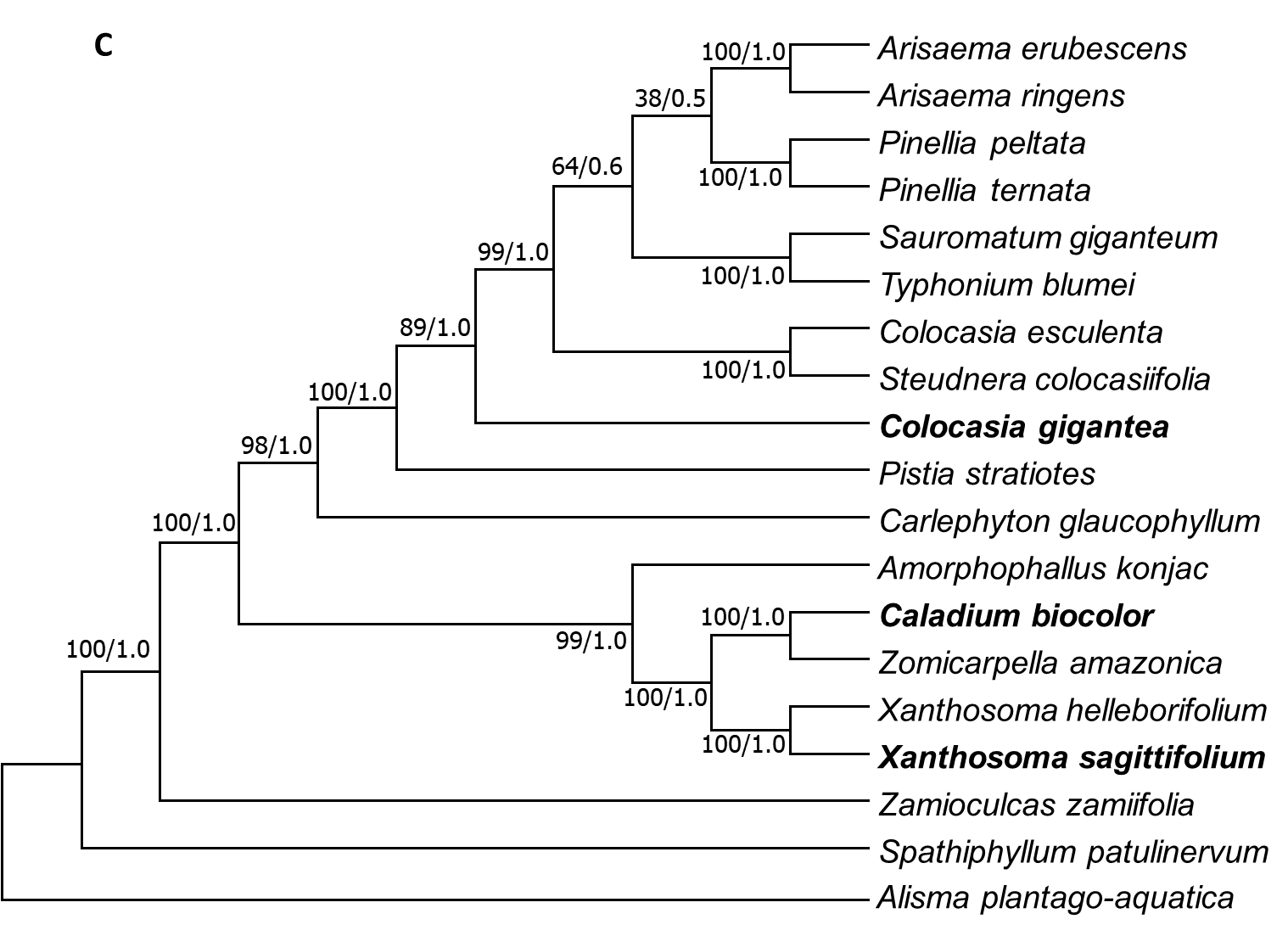

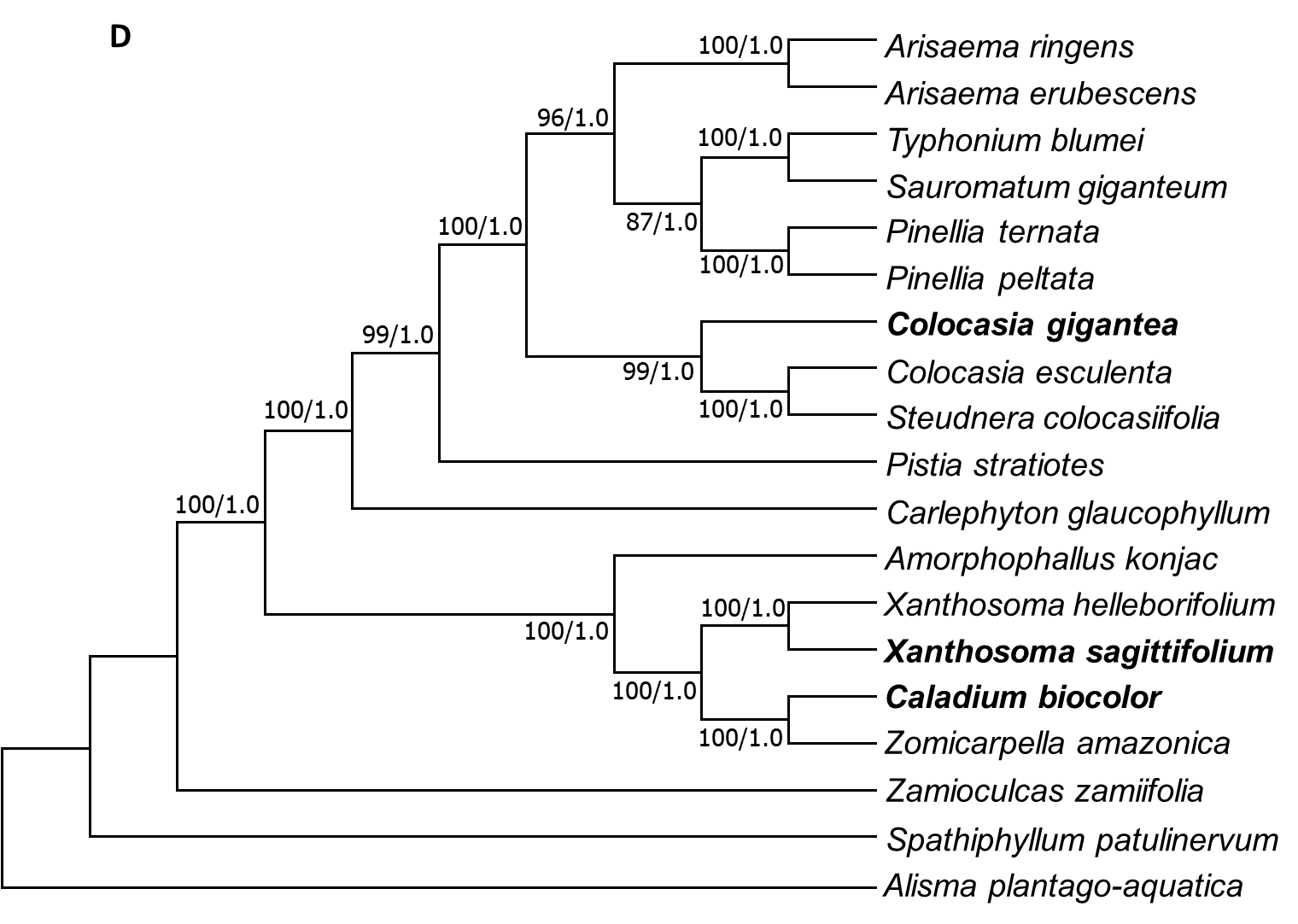


**Fig. S2.** Phylogenetic trees of the 17 Aroideae species based on the chloroplast genome by ML and BI analyses. The bootstrap values of ML and Bayesian posterior probabilities of BI analyses are shown beside the node of clades. A. Phylogenetic tree constructed using the CPG; B. Phylogenetic tree constructed using LSC region; C. Phylogenetic tree constructed using IR region; D. Phylogenetic tree constructed using SSC region. *Spathiphyllum patulinervum* and *Alisma plantago-aquatica* were used as the outgroups. *C. gigantea*, *C. bicolor* and *X. sagittifolium* were marked in bold characters.


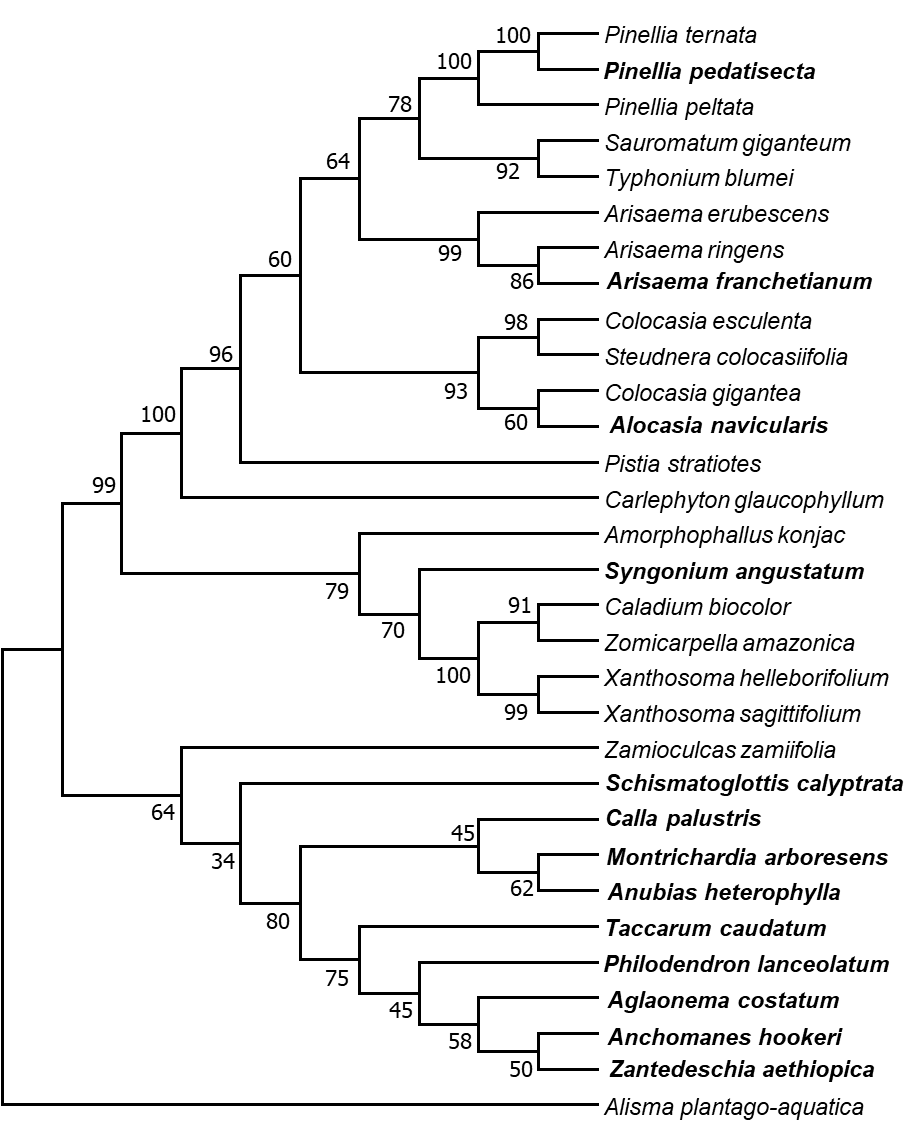


**Fig. S3.** Phylogenetic trees of the 30 Aroideae species based on the chloroplast genome by ML. Phylogenetic tree constructed using the atpH-atpI + psaC-ndhE + trnS-trnG, *Alisma plantago-aquatica* were used as the outgroups. 13 published chloroplast genomes of Aroideae species were marked in bold characters.
